# Nano‐hydroxyapatite preparation for the remineralization of primary tooth enamel surface subjected to liquid medication: An observational study

**DOI:** 10.1002/hsr2.1188

**Published:** 2023-04-11

**Authors:** Noor M. Hassan, Zainab J. Jafar, Mohammed H. Abdul Latif

**Affiliations:** ^1^ Department of Pedodontics and Preventive Dentistry, College of Dentistry University of Baghdad Baghdad Iraq; ^2^ Chemistry Department, College of Education for Pure Science Ibn‐ Al‐Haitham University of Baghdad Baghdad Iraq

**Keywords:** atomic force microscope, liquid medications, nano‐hydroxyapatite, primary tooth enamel surface, remineralization

## Abstract

**Background and Aims:**

Acid‐induced demineralization may be caused by the consumption of liquid medications routinely administered to children. Therefore, different remineralizing agents, such as fluorides and nano‐hydroxyapatite, have been added to oral care products to remineralize erosive lesions. This study was conducted to compare the effectiveness of 1% nano‐hydroxyapatite suspension and 2% sodium fluoride solution on the surface texture of primary teeth enamel previously exposed to liquid drugs.

**Methods:**

Thirty posterior primary teeth were extracted and grouped depending on the remineralizing agent used: (A) nano‐hydroxyapatite and (B) sodium fluoride. Groups A and B were subjected to liquid medication in two subgroups: cephalexin (cephalexin monohydrate) and ParAzar (acetaminophen), followed by remineralization with 1% nano‐hydroxyapatite suspension and 2% sodium fluoride solution. An atomic force microscope was used to analyze the surface texture of the primary tooth enamel by measuring the roughness and waviness parameters of the tested surfaces. The examination was performed at baseline, 7 days after exposure to drugs, and after exposure to remineralizing agents.

**Results:**

A significant difference in enamel surface roughness between nano‐hydroxyapatite‐treated surfaces and sodium fluoride‐treated surfaces was observed. A significant change was observed in the surface waviness of the primary enamel surface of sodium‐fluoride‐treated teeth that were subjected to cephalexin.

**Conclusion:**

Routine use of liquid medications could have a negative impact on primary enamel surface topography, because the primary tooth enamel is less mineralized than permanent tooth enamel. Our results show that compared with a 2% sodium fluoride solution, a 1% nano‐hydroxyapatite suspension can be used for remineralizing and restoring defects of the enamel surface of primary teeth following exposure to liquid medication, thus reinforcing dental tissues with higher efficacy.

## 1 INTRODUCTION

Various drugs affect dental hard tissues leading to enamel hypomineralization and erosion, which is especially concerning for deciduous dentition. Since the occurrence of these changes is progressive and slow, early diagnosis of their clinical and etiological factors is key to preventing their damaging effects.^1^

The use of antibiotic and analgesic medications in children is unavoidable. Additionally, their consequent harmful effects on oral structures are exacerbated by their long‐term use, leading to sustained negative outcomes.^2^

Acid exposure leads to the progressive dissolution of minerals, which results in the loss of dental hard tissues. Therefore, the best way to prevent tooth erosion is to minimize the amount and frequency of contact with acidic syrups and suspensions routinely used by children for medical purposes, thereby increasing acid resistance by the remineralization of the enamel surface.^3^

The remineralization potential of hydroxyapatite nanoparticles has evolved as they act as fillers repairing small holes in the tooth structure.^4^ They have been safely incorporated in dental care products such as mouth rinses and toothpastes and are beneficial in promoting enamel remineralization and treating dental hypersensitivity by replacing the lost minerals and restoring defects, thus reinforcing dental tissues.^5^

The present study aimed to compare the efficacy of routinely used sodium fluoride (NaF) solution with newly prepared nano‐hydroxyapatite (N‐HAp) suspension on the surface texture of primary tooth enamel subjected to erosion by liquid antibiotics and analgesics, using an atomic force microscope (AFM).

## 2 MATERIALS AND METHODS

### 2.1 Sample collection

This observational study was conducted from January 20, 2019, to March 3, 2020, at the Department of Pedodontics and Preventive Dentistry of the College of Dentistry, University of Baghdad. Ethical approval (No. 15) was obtained from the ethical committee of the College of Dentistry, University of Baghdad on January 8, 2019.

The study samples were obtained from 30 posterior primary teeth that were extracted from children aged 3–6 years for reasons such as mobility, nonrestorable badly carious teeth, or tooth root resorption. The selected teeth were extracted at the Department of Pedodontics and Preventive Dentistry at the College of Dentistry. Consent for tooth extractions for research purposes was obtained from the children's parents.

### 2.2 Sample preparation, grouping, and remineralizing agents used

The teeth were stored in distilled water at room temperature in universal glass tubes. The distilled water was replaced weekly to prevent bacterial colonization until the study was undertaken.

Before use, the teeth selected for sample preparation were polished with nonfluoridated pumice using a rubber cup of a low‐speed handpiece to remove any remnant debris on the tooth surface. The samples were then washed with distilled water and dried using cotton pads.

Primary tooth blocks of 4‐mm width and 2‐mm thickness were prepared using a micromotor handpiece. All surfaces were covered with adhesive tape excluding the surface to be tested for its identification and accurate measurement of changes.

Thereafter, the prepared samples were divided equally to Groups A and B and subdivided according to the type of liquid medication (LM) used to induce demineralization; the first subgroup was exposed to the antibiotic cephalexin (250 mg/5 mL), and the second subgroup was exposed to the analgesic ParAzar‐acetaminophen (120 mg/5 mL). All of these drugs were prescribed by medical professionals working at the Iraqi Ministry of Health to be used for the treatment of children attending their healthcare facilities.

Experimental samples were immersed in LM and vibrated every 8 h for 1 min. This was repeated three times a day for 7 days corresponding to the dose of each drug.^6^ During intervals, artificial saliva was used to store the samples to mimic the oral environment. The saliva was replaced daily to maintain ionic balance and acidity.^7^, ^8^ Artificial saliva was prepared by dissolving different compounds in varying concentrations, such as sodium carboxymethyl cellulose, calcium chloride, potassium phosphate, and NaF, in deionized water. The pH of the prepared saliva sample was adjusted to 7.^9^

Afterward, samples in all groups were subjected to the following remineralizing preparations:

‐ Group A samples were remineralized with 1% N‐HAp suspension once for 5 s.
‐ Group B samples were remineralized with 2% NaF solution once for 1 min.

#### 2.2.1 Preparation of N‐HAp suspension

A 1% N‐HAp suspension was prepared at the Chemistry Department, College of Education‐Ibn Al‐Haitham, University of Baghdad by suspending (0.05 g) of N‐HAp powder [Ca_10_(PO_4_)_6_(OH)_2_, SkySpring Nanomaterials, Inc.] in a small quantity (0.1 mol/L) of phosphoric acid (H_3_PO_4_) in a 5 mL volumetric flask. Being a weak acid (pH is approximately 1.5), it would preserve the stability of N‐HAp powder. Subsequently, calcium hydroxide [Ca(OH)_2_], which is a strong base, was added dropwise to neutralize the solution to adjust the pH to 7.

Finally, the prepared solution was diluted in distilled water and mixed well after being placed in an ultrasonic device (POWER SONIC 405‐Microprocess Controlled Bench‐top Ultrasonic device) in a water bath at 40°C for 3 h to obtain a colloidal suspension.

#### 2.2.2 Preparation of NaF solution

A 2% NaF solution was prepared by dissolving 2 g of NaF powder in 100 mL of distilled water in a volumetric flask.^10^

### 2.3 Measurement of changes in primary tooth enamel surface texture

The topographical changes of primary tooth enamel surface were assessed by measuring changes in surface texture (roughness and waviness) of the samples using an AFM (Ntegra/Russian Federation).

AFM is a type of scanning probe microscope that provides three‐dimensional topographic analysis at a high atomic resolution.^11^ It is operated by attaching a probe with a sharp tip at the end of a flexible cantilever and scanning it across the sample surface.^12^

The measurements were performed using the tapping mode of the AFM to display topographical images of the surface, which were taken at a 20 × 20‐µm scan size to form a three‐dimensional image of each sample obtained by measuring the surface height at each pixel or point in an image, represented by high areas (peaks) and low areas (valleys). Surface texture was quantified as roughness and waviness parameters, root mean square roughness (Rq) and average waviness (Wa), respectively, measured in nanometers.^13^ The Rq and Wa values were recorded before the experiment, 1 week after exposure to the LM, and after exposure to remineralizing agents (N‐HAp and NaF).

Roughness is typically defined as the irregularities of a surface that are closely spaced and measured using the vertical spacing from the ideal surface form. When the spacing is large, the surface is rough, and when it is small, the surface is smooth. Waviness is a wider prospect of roughness because it is defined as irregularities, whereby the average spacing between waviness peaks is greater than the roughness sampling length.^14^

### 2.4 Pilot study

A pilot study was conducted using a few primary teeth samples. The teeth were evaluated at three time points: baseline, 7 days after 1 min exposure to the LM, and after exposure to different concentrations of N‐HAp. The AFM was used to measure changes in the surface texture (Rq, Wa).

Initially, a 5% N‐HAp suspension was used. The first sample was immersed in the prepared suspension for 1 min, twice a day for 3 days. The second sample was immersed in 5% N‐HAp suspension for 1 min only once. Subsequently, the N‐HAp concentration was reduced to 2% for the third sample, which was also immersed once for 1 min. Finally, the fourth sample was immersed in 1% N‐HAp suspension once for 5 s. There was a significant increase in Rq and Wa values after exposure to different concentrations of N‐HAp when compared to those after exposure to LM.

The lowest concentration (1%) and time (5 s) required for treatment were necessary because the study samples were extracted from children aged 3–6 years, suggesting that such professional treatment for that age group should utilize minimum concentrations, keeping in mind the rationale of accidental swallowing and inability of the child to keep the solution in the mouth for a long time without being swallowed.

### 2.5 Statistical analysis

Analyses were performed using the Statistical Package for Social Sciences (version 21) software, including descriptive and inferential statistics.

For descriptive statistics, data for each group and subgroup are presented as means and standard deviations.

For inferential statistics, the Shapiro–Wilk test was used to analyze whether data were normally distributed. The Rq and Wa values were compared at different time points among the studied groups and subgroups using the two‐way repeated measurements analysis of variance (RM‐ANOVA). Pairwise comparisons (post hoc Bonferroni test) were used to detect significance between subgroups at different time points. Bar charts were calculated as error bars with 95% confidence intervals.

The level of significance was set at a probability value (*p*) of 0.05 for all tests.

- *p* ≥ 0.05—Nonsignificant.
- *p* < 0.05—Significant.

## 3 RESULTS

**Figure 1:**
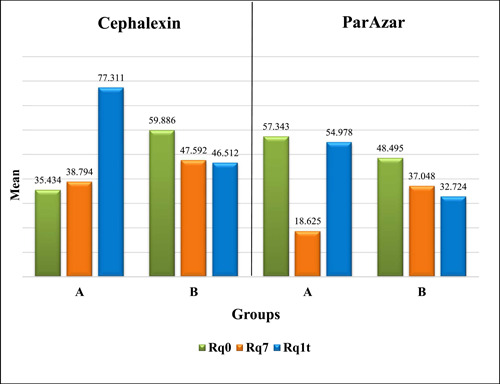
Comparison of root mean square roughness at different periods. Rq0, baseline root mean square roughness; Rq7, root mean square roughness after 7 days of exposure to Cephalexin and Parazar; Rq1t, root mean square roughness after one‐time exposure to N‐HAp and NaF. A, N‐HAp group; B, NaF group.

The results of the Shapiro–Wilk test revealed that the data were normally distributed. The comparison of mean Rq values between Groups A and B using the 2‐way RM‐ANOVA revealed that there was a significant difference in Group A for cephalexin and ParAzar subgroups (*p* = 0.003 and 0.004, respectively), as shown in Table 1 and Figure 1.

**Table 1:** Intergroup comparison of root mean square roughness at different periods.

Groups	Subgroups	Rq0^a^		Rq7^b^		Rq1t^c^		*p* Value
		Mean	SD	Mean	SD	Mean	SD	
A	Cephalexin	35.434	18.727	38.794	16.364	77.311	35.603	0.003
	ParAzar	57.343	16.753	18.625	8.108	54.978	30.323	0.004
B	Cephalexin	59.886	20.979	47.592	19.668	46.512	22.749	0.267
	ParAzar	48.495	25.694	37.048	14.047	32.724	15.971	0.224

*Note*: *p* < 0.05—significant; *p* ≥ 0.05—nonsignificant.

Abbreviations: LM, liquid medication; NaF, sodium fluoride; N‐HAp, nano‐hydroxyapatite.

^a^ Baseline root mean square roughness.

^b^ Root mean square roughness after 7 days of exposure to LM.

^c^ Root mean square roughness after one‐time exposure to N‐HAp (A) and NaF (B).

Results of the post hoc Bonferroni test of Rq for group A at different time points showed that there were significant differences between remineralizing agents and LM exposure periods; mean Rq values increased after using N‐HAp, and there was a significant difference between baseline and remineralization periods for cephalexin, and between baseline and ParAzar exposure periods (Table 2).

**Table 2:** Pairwise comparisons of root mean square roughness within Group A between subgroups.

Bonferroni test					
Groups	Subgroups	Periods		Mean difference	*p* Value
A (N‐HAp)	Cephalexin	Rq0^a^	Rq1t^b^	−41.877	0.002
			Rq7^c^	−3.360	1.000
		Rq1t^b^	Rq7^c^	38.516	0.030
	ParAzar	Rq0^a^	Rq1t^b^	2.365	1.000
			Rq7^c^	38.718	0.002
		Rq1t^b^	Rq7^c^	36.353	0.043

*Note*: *p* < 0.05—significant; *p* ≥ 0.05—nonsignificant.

Abbreviations: LM, liquid medication; N‐HAp, nano‐hydroxyapatite.

^a^ Baseline root mean square roughness.

^b^ Root mean square roughness after one‐time exposure to N‐HAp.

^c^ Root mean square roughness after 7 days of exposure to LM.

**Figure 2:**
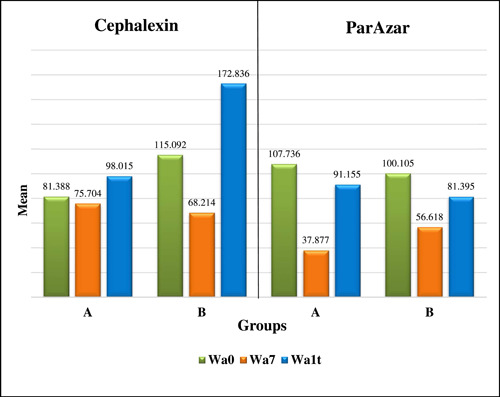
Intergroup comparison of average waviness among Cephalexin and Parazar subgroups at different periods. Wa0, baseline average waviness; Wa7, average waviness after 7 days of exposure to Cephalexin and Parazar; Wa1t, average waviness after one‐time exposure to N‐HAp and NaF. A, N‐HAp group; B, NaF group.

Comparison of the Wa for Groups A and B at different time points revealed that there was a significant difference in Group B for the cephalexin subgroup (Figure 2), while there were nonsignificant differences in Group A for both subgroups (Table 3).

**Table 3:** Average waviness compared at different periods for Groups A and B by subgroups.

Groups	Subgroups	Wa0^a^		Wa7^b^		Wa1t^c^		*p* Value
		Mean	SD	Mean	SD	Mean	SD	
A	Cephalexin	81.388	41.260	75.704	30.008	98.015	58.415	0.786
	ParAzar	107.736	71.592	37.877	34.016	91.155	60.022	0.073
B	Cephalexin	115.092	103.08	68.214	40.244	172.836	138.773	0.010
	ParAzar	100.105	78.015	56.618	33.320	81.395	70.454	0.407

*Note*: *p* < 0.05—significant, *p* ≥ 0.05—nonsignificant.

Abbreviations: LM, liquid medication; NaF, sodium fluoride; N‐HAp, nano‐hydroxyapatite.

^a^ Baseline average waviness.

^b^ Average waviness after 7 days of exposure to LM.

^c^ Average waviness after one‐time exposure to N‐HAp (A) and NaF (B).

Pairwise comparison of Wa for group B in the cephalexin subgroup showed a significant difference (*p* = 0.011) between the one‐time NaF and 7‐day cephalexin exposure periods, as exhibited in Table 4.

**Table 4:** Pairwise comparison of average waviness for Group B by periods.

Bonferroni test					
Groups	Subgroups	Periods		Mean difference	*p* Value
B (NaF)	Cephalexin	Wa0^a^	Wa1t^b^	−57.744	0.710
			Wa7^c^	46.877	0.633
		Wa1t^b^	Wa7^c^	104.621	0.011

*Note*: *p* < 0.05—significant; *p* ≥ 0.05—nonsignificant.

Abbreviations: LM, liquid medication; NaF, sodium fluoride; N‐HAp, nano‐hydroxyapatite.

^a^ Baseline average waviness.

^b^ Average waviness after one‐time exposure to NaF.

^c^ Average waviness after 7 days of exposure to LM.

**Figure 3:**
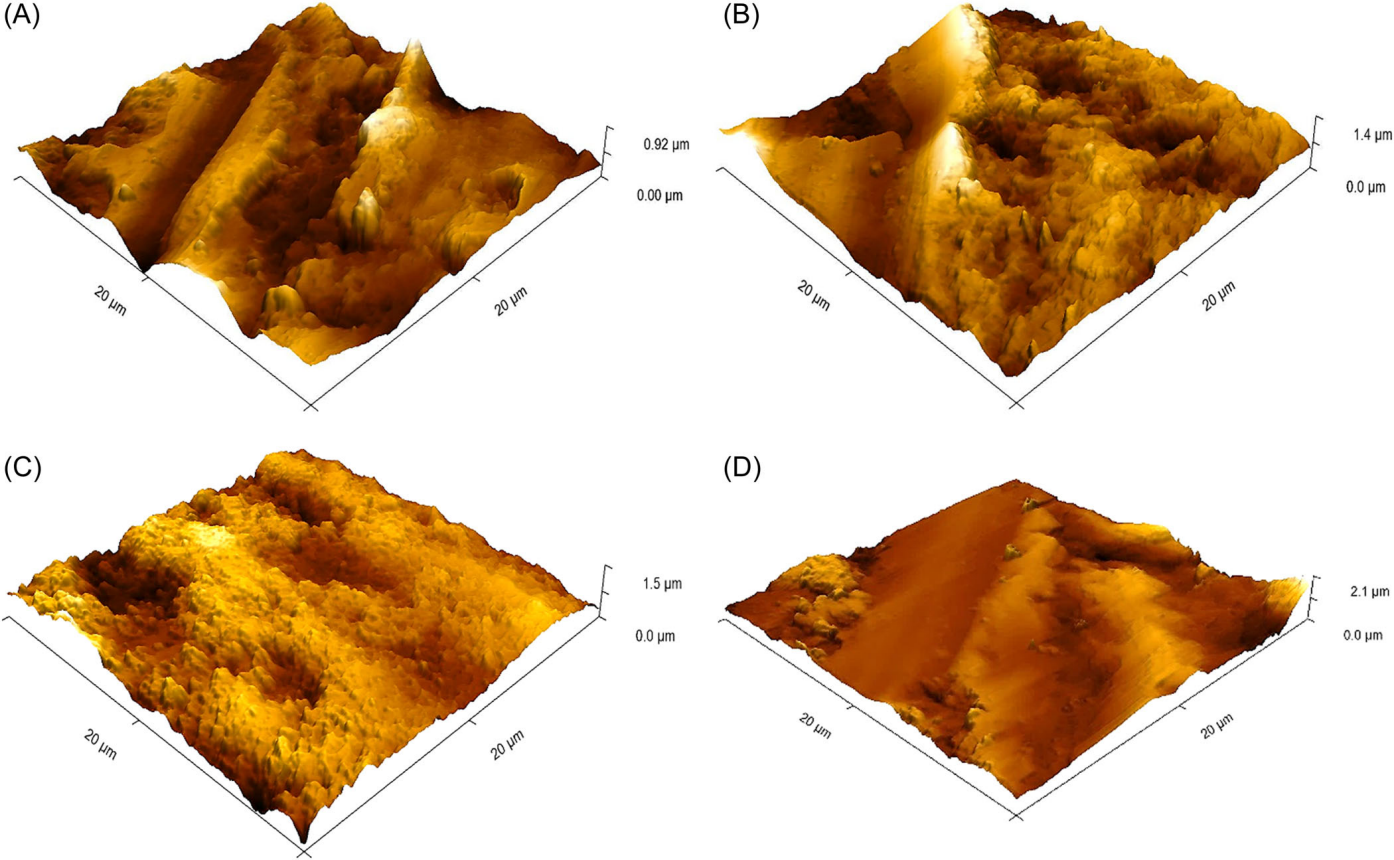
Three‐dimensional images of outer enamel surface under AFM (20 × 20 µm). (A) Baseline period. (B) After exposure to LM. (C) After remineralization with N‐HAp. (D) After remineralization with NaF. AFM, an atomic force microscope; LM, liquid medication; NaF, sodium fluoride; N‐HAp, nano‐hydroxyapatite.

Topographical observation of the primary enamel surfaces using the AFM revealed a smooth and intact surface with few globules, which represented the hydroxyapatite crystals on the tooth before the experiment began (Figure 3A), while a significant increase was observed in surface valleys that appeared as porosities after a week of exposure to the LM (Figure 3B). Comparison of the results of both remineralizing agents used in this study showed that the Group A samples had an increased occurrence of homogenous surface peaks, which formed a protective layer of clusters of crystals covering the enamel surface remineralized with 1% N‐HAp suspension (Figure 3C). In contrast, the enamel surfaces of the Group B samples displayed an alternating pattern of nonuniform peaks and valleys, which appeared as a layer of crystals of varying sizes and shapes deposited on the enamel surface remineralized with 2% NaF solution (Figure 3D).

## 4 DISCUSSION

Routine use of LM such as antibiotics and analgesics may have an impact on the surface texture and topography of primary teeth, because the enamel of primary teeth is thinner and less mineralized than that of permanent teeth.^15^ Accordingly, the damaging effect of the LM used in this study was associated with acidic attacks on the enamel surface with subsequent mineral loss caused by cephalexin and ParAzar used for 1 week. Remineralizing agents comprising N‐HAp and NaF were tested to restore the demineralized enamel surface affected by erosion.

Changes in the surface roughness and waviness of the study samples were compared. In terms of surface roughness, the mean Rq values of group A increased after the LM exposure period for both subgroups following N‐HAp remineralization. This could be due to the degradation of the enamel surface, in which surface peaks indicating the roughness were demineralized because of the acids included in the compositions of the medications used (cephalexin pH = 4.8, ParAzar pH = 4.9). Thus, the mean Rq values decreased after 7 days of exposure to LM. These results are similar to those of the study by Valinoti et al., which showed that acidic medications such as antibiotics could modify enamel surface morphology, considering their long‐term usage. In addition, those drugs could cause erosion owing to their acidic formulations, availability of buffering agents, and lack or low concentrations of fluoride, calcium, and phosphate ions.^16^

Regarding analgesics, the results of the present study are in accordance with those of the study by Saeed et al., which showed that nearly all analgesics had pH values ≤ 5.5, thus promoting enamel dissolution. This pH value is critical because it usually increases the cariogenic and erosive potential of these drugs.^17^

The significant increase in surface roughness after remineralization may be expressed by the bioactivity and biocompatibility of N‐HAp, because these properties protect the enamel against erosion. Atomic proportion and surface area increase with reducing particle size; therefore, N‐HAp can be efficiently adsorbed by the enamel surface providing a large amount of calcium and phosphate ions promoting the growth of existing crystals, resulting in a rougher surface.^18^, ^19^ In addition, the N‐HAp used in the group A samples formed uniform clusters of crystals over enamel defects, thereby preventing demineralization.^20^ This result was compatible with that of the AF microscopy.

Regarding waviness, the significant increase in Wa values after using the 2% NaF solution for the cephalexin subgroup can be explained by the formation of a nonuniform globular calcium fluoride layer composed of variable crystal shapes and sizes, resulting in larger and more distinct irregularities.

Under the AFM, samples remineralized with 1% N‐HAp suspension showed the deposition of a homogenous layer of nearly uniform N‐HAp crystals, leading to fewer or smaller irregularities on the enamel surface, which explains the lower mean Wa values for Group A compared to those of Group B.

## 5 CONCLUSION

Our results showed that following exposure to LM, 1% N‐HAp suspension had a greater remineralization‐promoting ability on primary tooth enamel surface compared to that of 2% NaF solution. Additionally, the 1% N‐HAp suspension formed a protective layer on the enamel surface.

## AUTHOR CONTRIBUTIONS

**Noor M. Hassan**: Data curation; formal analysis; investigation; project administration; resources; software; writing—original draft; writing—review and editing. **Zainab J. Jafar**: Conceptualization; data curation; formal analysis; methodology; resources; supervision; validation; visualization. **Mohammed H. Abdul Latif**: Conceptualization; methodology; resources; validation; visualization.

## CONFLICT OF INTEREST STATEMENT

The authors declare no conflict of interest.

## TRANSPARENCY STATEMENT

The lead author Noor Mohammed Hassan affirms that this manuscript is an honest, accurate, and transparent account of the study being reported; that no important aspects of the study have been omitted; and that any discrepancies from the study as planned (and, if relevant, registered) have been explained.

## Data Availability

All authors have read and approved the final version of the manuscript. The corresponding author (Noor M. Hassan) has full access to all of the data in this study and takes complete responsibility for the integrity of the data and accuracy of the data analysis.
